# Evaluation of CircRNA Sequence Assembly Methods Using Long Reads

**DOI:** 10.3389/fgene.2022.816825

**Published:** 2022-02-14

**Authors:** Jingjing Zhang, Md. Tofazzal Hossain, Weiguo Liu, Yin Peng, Yi Pan, Yanjie Wei

**Affiliations:** ^1^ University of Chinese Academy of Sciences, Beijing, China; ^2^ Centre for High Performance Computing, Shenzhen Institutes of Advanced Technology, Chinese Academy of Sciences, Shenzhen, China; ^3^ School of Software, Shandong University, Jinan, China; ^4^ Guangdong Key Laboratory for Genome Stability and Disease Prevention and Regional Immunity and Diseases, Department of Pathology, Shenzhen University School of Medicine, Shenzhen, China; ^5^ CAS Key Laboratory of Health Informatics, Shenzhen Institutes of Advanced Technology, Chinese Academy of Sciences, Shenzhen, China

**Keywords:** circRNA, full-length sequences, short reads, long reads, assembly

## Abstract

The functional study on circRNAs has been increasing in the past decade due to its important roles in micro RNA sponge, protein coding, the initiation, and progression of diseases. The study of circRNA functions depends on the full-length sequences of circRNA, and current sequence assembly methods based on short reads face challenges due to the existence of linear transcript. Long reads produced by long-read sequencing techniques such as Nanopore technology can cover full-length sequences of circRNA and therefore can be used to evaluate the correctness and completeness of circRNA full sequences assembled from short reads of the same sample. Using long reads of the same samples, one from human and the other from mouse, we have comprehensively evaluated the performance of several well-known circRNA sequence assembly algorithms based on short reads, including circseq_cup, CIRI_full, and CircAST. Based on the F1 score, the performance of CIRI-full was better in human datasets, whereas in mouse datasets CircAST was better. In general, each algorithm was developed to handle special situations or circumstances. Our results indicated that no single assembly algorithm generated better performance in all cases. Therefore, these assembly algorithms should be used together for reliable full-length circRNA sequence reconstruction. After analyzing the results, we have introduced a screening protocol that selects out exonic circRNAs with full-length sequences consisting of all exons between back splice sites as the final result. After screening, CIRI-full showed better performance for both human and mouse datasets. The average F1 score of CIRI-full over four circRNA identification algorithms increased from 0.4788 to 0.5069 in human datasets, and it increased from 0.2995 to 0.4223 in mouse datasets.

## Introduction

Only recently has circular RNA (circRNA) appeared as a hot research topic since it was first discovered in the 1970s (Sanger et al., 1976; Arnberg et al., 1980; Kos et al., 1986). Different from linear RNAs, the special covalent circular structure of circRNA is formed by back splicing (Jeck et al., 2013). Identifying the back splice sites is the most important factor for circRNA identification from the sequencing reads (Kristensen et al., 2019). Based on sequencing data, various identification algorithms were developed, such as find_circ (Memczak et al., 2013), KNIFE (Szabo et al., 2015), CIRI (Y. Gao et al., 2015), and PCirc (Yin et al., 2021), some of which require annotation information of genome sequences to improve identification sensitivity and reduce the false discovery rate (FDR) (Memczak et al., 2013; Baruzzo et al., 2017).

As more and more circRNAs were discovered in animals and plants in recent years (Glažar et al., 2014; J.; Zhang et al., 2020a), new functions of circRNAs in the organism have also been discovered. Acting as micro RNA (miRNA) sponge is mostly studied for circRNAs, and circRNAs regulate expression of miRNA target gene indirectly (Piwecka et al., 2017). Hansen *et al* found that exonic circRNA CDR1as can bind with miR-671, which can degrade CDR1as mediated by AGO (Hansen et al., 2013), and the binding sites are highly conserved. In addition, circRNAs can also interact with RNA binding proteins as endogenous competitive RNA (S. Zheng et al., 2021). The gene *muscleblind* (*MBL*) of Drosophila can encode MBL protein as a transcript factor, and MBL regulates the dynamic balance of circular transcript (circRNA circMbl) and linear transcript (Ashwal-Fluss et al., 2014). Although circRNAs were considered to be noncoding RNAs (Qu et al., 2015), some circRNAs have been found to translate proteins (Shi et al., 2020). For example, circRNA circPINT can translate into protein PINT-87aa for inhibiting malignant glioma (M. Zhang et al., 2018). Another circRNA, circE7, derived from oncogenic human papilloma viruses (HPVs), is found to produce E7 oncoprotein with modified N6-methyladenosine (m6A) (Zhao et al., 2019).

For the study of circRNA functions, sequence information is vital. Due to its special structure, it is difficult to obtain correct and complete sequences of circRNAs (full-length sequences) directly. Reconstruction of circRNAs full-length sequences was effected by linear transcripts (Szabo & Salzman, 2016). Computational tools such as circseq_cup (Ye et al., 2017), CIRI-full (Y. Zheng et al., 2019), and CircAST (Wu et al., 2019) were developed to assemble full-length sequences for circRNAs according to short reads (next-generation sequencing data and RNA-Seq data).

circseq_cup predicts circRNAs and constructs full-length sequences based on paired-end (PE) short reads. This method first relies on an alignment software (TopHat-Fusion, STAR-Fusion, or segemehl (Kim & Salzberg, 2011; Dobin et al., 2013; Hoffmann et al., 2014)) to identify fusion junction sites. The construction of the virtual reference sequence concatenates sequences between fusion junction sites. Full-length sequences of circRNAs were assembled by PE reads that could align to the middle of virtual reference sequences. Then, some criteria were used to filter out false-positive circRNAs, such as sequences supported by less than two pairs of PE reads. CIRI-full introduces a new feature named reverse overlap (RO) for assembling candidate circRNA sequences. Back-splice junctions (BSJs) are PE reads that are aligned to back splice sites which support the identification of circRNA. If RO reads or BSJ reads can cover all cirexons (circRNA’s exon) between back splice sites, the complete sequences of circRNA can be assembled by connecting the cirexons. Otherwise, a combined strategy based on both RO reads and BSJ reads were used to reconstruct circRNA full-length sequences. Performance improvement of CIRI-full relies on longer reads, such as longer than 250 bp. CircAST assembles circRNA full-length sequences with mapped fragments using a multiple splice graph model. Each transcript was represented by a directed acyclic graph (DAG), exons between back splice sites represent the nodes on the graph, and directed edges on the graph indicate the mapped reads mapped on these two different exons. Source node and sink node should be the exons mapped by the fragments of back splice reads of circular transcript. In addition, CircAST is an annotated-based method and shows better performance on shorter read lengths (from 75 bp to 125 bp). For all the software/methods, the correctness and completeness of the constructed circRNA sequences are difficult to evaluate. Assembly software based on short reads could only reconstruct full-length sequences for some circRNAs due to the interference of linear transcripts, and some assembled circRNA full-length sequences are false positive due to the same reason (X. Li et al., 2020).

Long-read sequencing, such as Nanopore sequencing, is capable of generating longer lengths, between 5,000 and 30,000 base pairs (van Dijk et al., 2018). Long reads have a higher error rate (10–15%), but these sequencing errors are randomly distributed; the rates can therefore be greatly reduced through the use of circular consensus sequencing (Larsen et al., 2014). This makes direct sequencing the full-length sequences of circRNAs possible since the length of most circRNAs under study is shorter than 5,000 bp (Z. Gao et al., 2019; J. Zhang et al., 2020b). Thus, by using long-read sequencing results of a sample, it is possible to evaluate the quality of assembled circRNA full-length sequences based on the short read sequencing results of the same sample.

In this study, we used three evaluation strategies (read alignment, CIRI-long, and isoCirc; see in Method) based on long reads to verify the quality of full-length sequences assembled based on short reads. In our results, each assembly algorithm showed its own advantage; in CircAST and circseq_cup, the precision was high but the sensitivity was low, whereas in CIRI-full, the precision was low but the sensitivity was high. CIRI-full performed better (F1 score, read alignment: 0.6348, CIRI-long: 0.4093, isoCirc: 0.5965) in *Homo sapiens* (human) datasets, while CircAST was the better performer in *Mus musculus* (mouse) datasets (F1 score, read alignment: 0.4112, CIRI-long: 0.4733, isoCirc: 0.3212). Among these assembly tools, CIRI-full assembled more circRNA full-length sequences with less than 57% of precision in human datasets, while circseq_cup and CircAST assembled few circRNAs full-length sequences with about 80% of precision in human datasets. After careful analysis, we have introduced a screening protocol that selects out exonic circRNAs with full-length sequences consisting of all exons between back splice sites as the final result. After screening, CIRI-full showed the best performance for both human and mouse datasets.

## Materials and Methods

### Data Collection

RNA-seq libraries (short reads; next-generation sequencing data) were downloaded from the Sequence Reads Archive (accession ID: SRR10612068, SRR10612069, and SRR10612070) and the National Genomics Data Center (https://bigd.big.ac.cn/gsa) (accession ID: CRR194214 and CRR194215). Nanopore libraries (long reads; third-generation sequencing data) were downloaded from the Sequence Reads Archive (accession ID: SRR10612050, SRR10612051, SRR10612052, SRR10612053, SRR10612054, and SRR10612055) and the National Genomics Data Center (accession ID: CRR194190, CRR194191, CRR194194, and CRR194195). Short reads and long reads from the same database were derived from the same experiment samples. Sequencing data downloaded from the SRA were all derived from the cultured HEK293 cells, and data downloaded from the NGDC were derived from adult mice. Table S1 provides a summary of all of the datasets. The reference genomes of human (GRCh38/hg38) and mouse (GRCm38/mm10) were downloaded from UCSC.

### Identification of circRNA and Recontruction of circRNA Full-Length Sequence Based on Short Reads

For analysis of short reads, sequencing reads were mapped to the genome using BWA (H. Li & Durbin, 2009), STAR (Dobin et al., 2013), and Tophat2 (Kim et al., 2013) with default parameters. Four tools, including CIRI2 (v2.0.6) (Y. Gao et al., 2018), CIRCexplorer2 (v2.3.5) (X. O. Zhang et al., 2014), circRNA_finder (v1.1) (Westholm et al., 2014), and find_circ (v1.2) (Memczak et al., 2013), were used for circRNA identification following the instructions of the software documentation. The identified circRNAs were selected with at least two back splice reads which were aligned to the circRNA junction sites.

Three pieces of software, circseq_cup, CIRI-full, and CircAST, were used for reconstruction of full-length sequences of circRNA with default parameters. Among them, CIRI-full and CircAST both require information of identified circRNA and sequencing reads as input, while circseq_cup only needs sequencing reads as input. Thus, for each short reads sequencing data, nine different results of full-length sequences are generated using different strategies, due to different combinations of identification algorithms and assembly algorithms.

### Evaluation of circRNA Full-Length Sequences Using Long Reads

Long reads data are a cluster of long-read sequences, most of which are longer than the full sequences of circRNA. One could assess whether circRNAs full-length sequences (most of their length <1,000 bp) that were reconstructed based on short reads are correct according to long-read sequences, given that both short reads and long reads are derived from the same samples.

**FIGURE 1:**
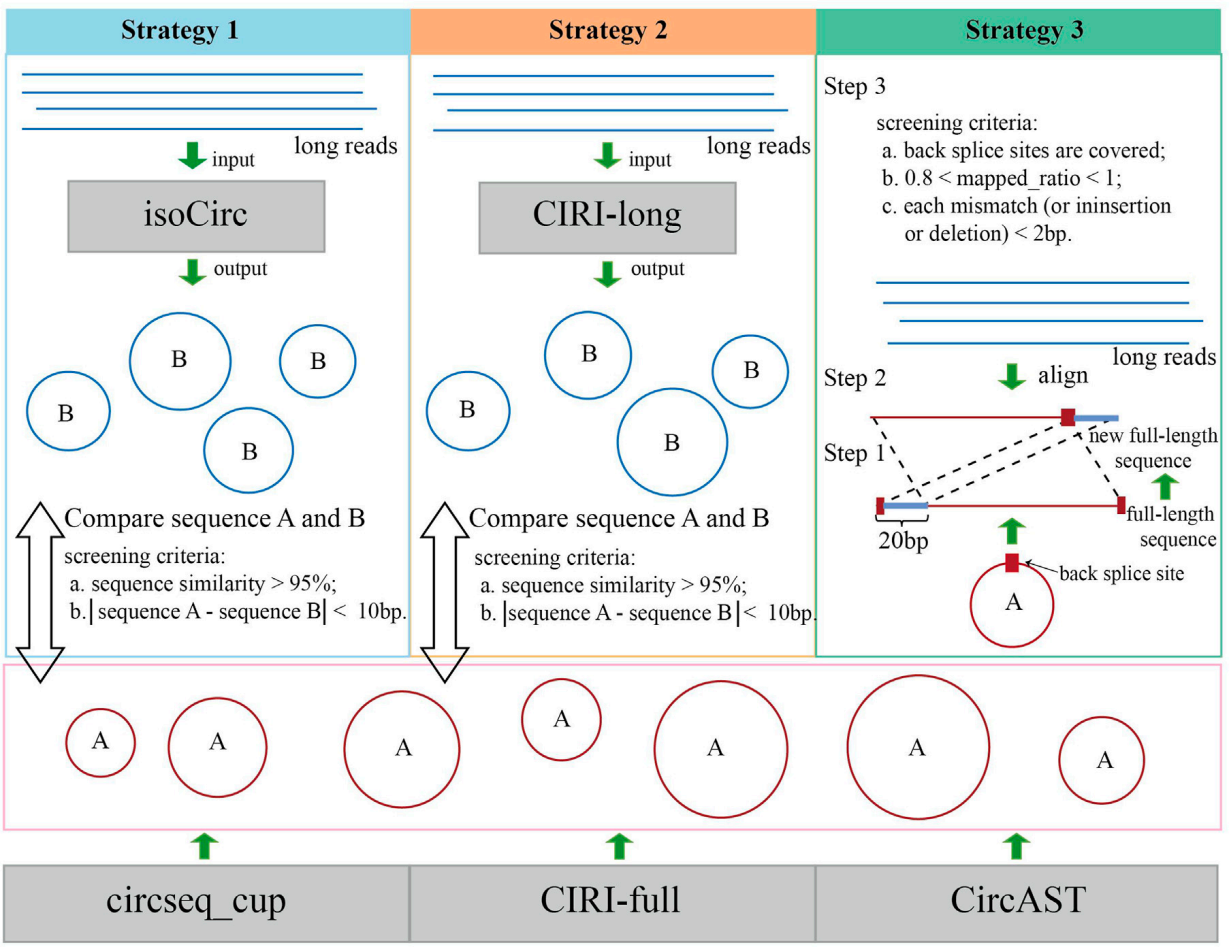
Evaluation of circRNA full-length sequences using long reads. Blue lines and circles **(B)** represent long reads or circRNAs identified using long reads; red lines and circles **(A)** represent assembled full-length sequence and circRNAs identified using short reads.

In this study, we have used three strategies based on long reads to evaluate the assembled circRNA full-length sequences using the short reads (Figure 1).

The correctness of the assembled sequence is evaluated using three strategies as shown in Figure 1. For strategy 1, isoCirc was used to determine the full-length circRNA isoforms from long reads. A sequence reconstructed from short reads was considered correct if it was similar to any one of the sequences of isoCirc results. Similarly, for strategy 2, CIRI-long was used to reconstruct full-length circRNA sequences using long reads.

Another evaluation strategy (strategy 3) used long reads to evaluate the correctness of the assembled circRNA sequences directly. Three main steps of strategy three were 1) we moved a 20 bp fragment on the upstream of the full-length sequence to the end of the full-length sequence, which forms a new full-length sequence with back splice sites; 2) long reads were mapped to the new full-length sequences of circRNAs using minimap2 (H. Li, 2018) with default parameters (-a); 3) for each alignment, mapped_ratio (M/L, where M is the number of mapped bases, and L is the number of bases of circRNA full-length sequences) was calculated; and 4) we discarded any alignment record with mapped_ratio >1 or <0.8, or they contained more than two bp mismatch, insertion, or deletion.

### Evaluation Metrics

In all evaluation strategies, full-length circRNAs that were verified correct by long reads were defined as true positives, while those not verified by long reads were defined as false positives. Full-length circRNAs were verified correct in other assembly strategies, but those not assembled in the currently evaluated assembly strategy were defined as false negatives. The assembly performance is assessed using precision, sensitivity, and F1 score and defined as follows:

$$precision=\frac{TP}{TP+FP}$$

$$sensitivity=\frac{TP}{TP+FN}$$

$$F1=\frac{2*precision*sensitivity}{precision+sensitivity}$$

where TP, FP, and FN are the number of true positives, false positives, and false negatives. F1 score weights precision and sensitivity equally and serves as a balanced metric to evaluate whether a tool achieves favorable precision and sensitivity simultaneously.

## Results

### Identification of circRNAs Based on Short Reads

**FIGURE 2:**
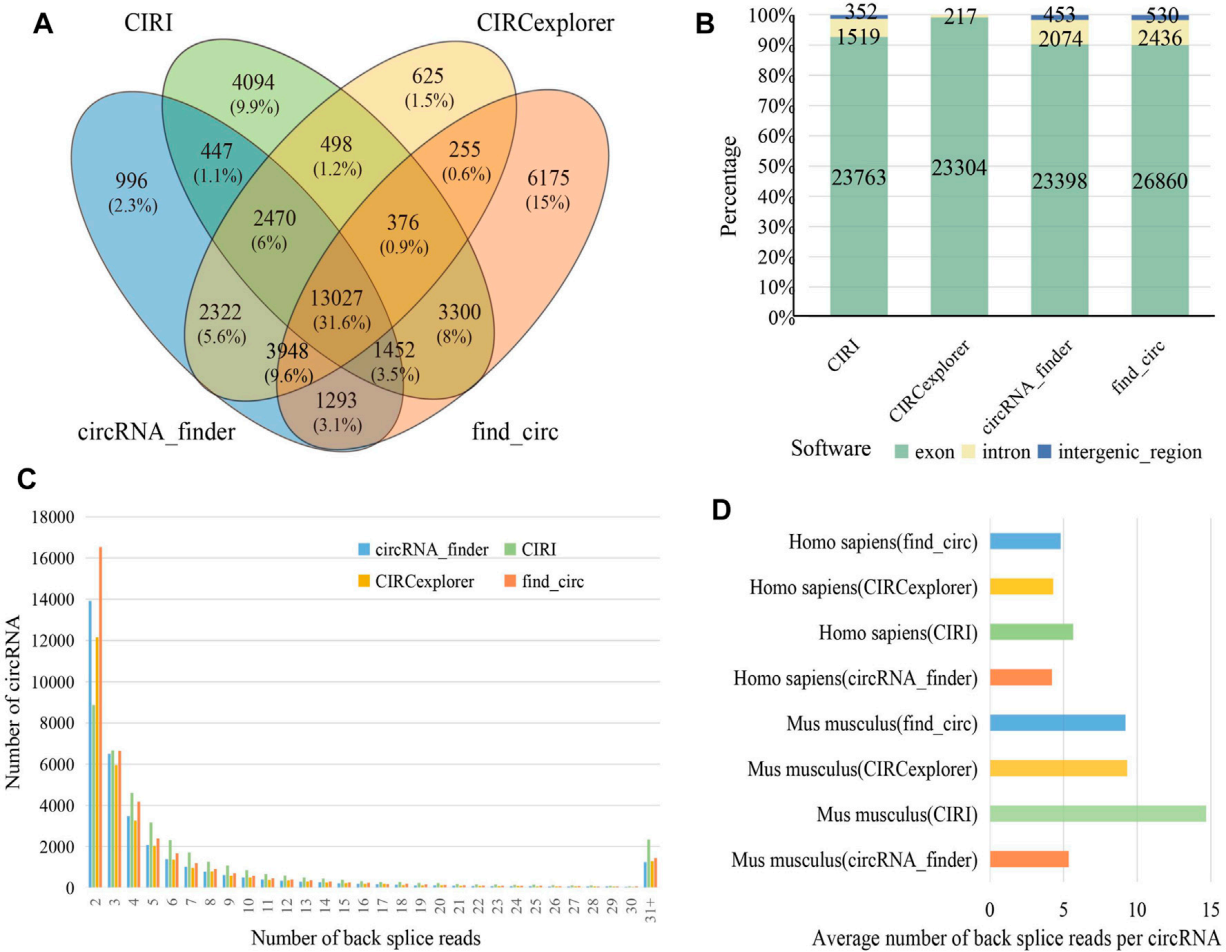
Identification and characterization of circRNAs. **(A)** Venn diagram depicting the overlap between the four different circRNA identification algorithms. **(B)** The percentage of different genomic origins of circRNA. **(C)** The distribution of back splice reads number in four identification algorithms. **(D)** Barplot showing average number of back splice reads per circRNA.

Several identification algorithms have been developed for circRNA identification based on short reads. In this study, we selected four algorithms to identify circRNA in human and mouse datasets, including CIRI, CIRCexplorer, circRNA_finder, and find_circ. Among the identified circRNAs, 13,027 (31.60%) were observed between all four algorithms (Figure 2A), while 11,890 (28.80%) were only found by a single algorithm. A total of 25,634 distinct circRNAs candidates were identified by CIRI, 23,763 (92.70%) of which were generated from exons, and the remaining were generated from introns or intergenic regions. For circRNA_finder and find_circ, 25,925 and 29,828 circRNAs were identified, respectively. Similarly, most of these circRNAs were derived from exons; only less than 10% were derived from introns and intergenic regions. However, among the circRNAs identified by CIRCexplorer, 23,304 (99.08%) were exonic, and 217 (0.92%) were intronic, but they were no intergenic circRNAs (Figure 2B). The number of circRNA candidates in each sample is shown in Table S2. By counting the number of back splice reads, 71.50% of circRNAs were supported by less than five back splice reads (Figure 2C), which agreed with the fact that circRNAs usually showed lower expression than linear transcripts (X. Li et al., 2018). CIRI produced a larger average number of back splice reads per circRNA in human and mouse than other algorithms (Figure 2D). In our results, more circRNAs were identified from mouse than human (Table S2), and circRNAs in mouse were supported by more back splice reads than in human (Figure 2D); these phenomena can be attributed to longer reads length (human: 101 bp and mouse: 151 bp) and greater sequencing depth of mouse datasets (Supplementary Table S1).

### Reconstruction of circRNA Full-Length Sequences Using Short Reads

Full-length sequences are important to analyze the function of circRNAs, such as miRNA sponges, RBP sites, and expression. Three popular methods, circseq_cup, CircAST, and CIRI-full, were used in this study for reconstructing full-length sequences of circRNA for short reads datasets.

As shown in Figrue 3 (A and B), less than 5% of the full-length circRNAs (circRNA that has the assembled full-length sequence) were common among all the three assembly tools for human and mouse datasets, and more than 95% of the reconstructed sequences of these pieces of software/methods were different. Thus, it is difficult for experimental biologists to select the circRNA sequences, and the functional study of circRNAs could be unreliable due to the wrongly selected circRNA sequences.

**FIGURE 3:**
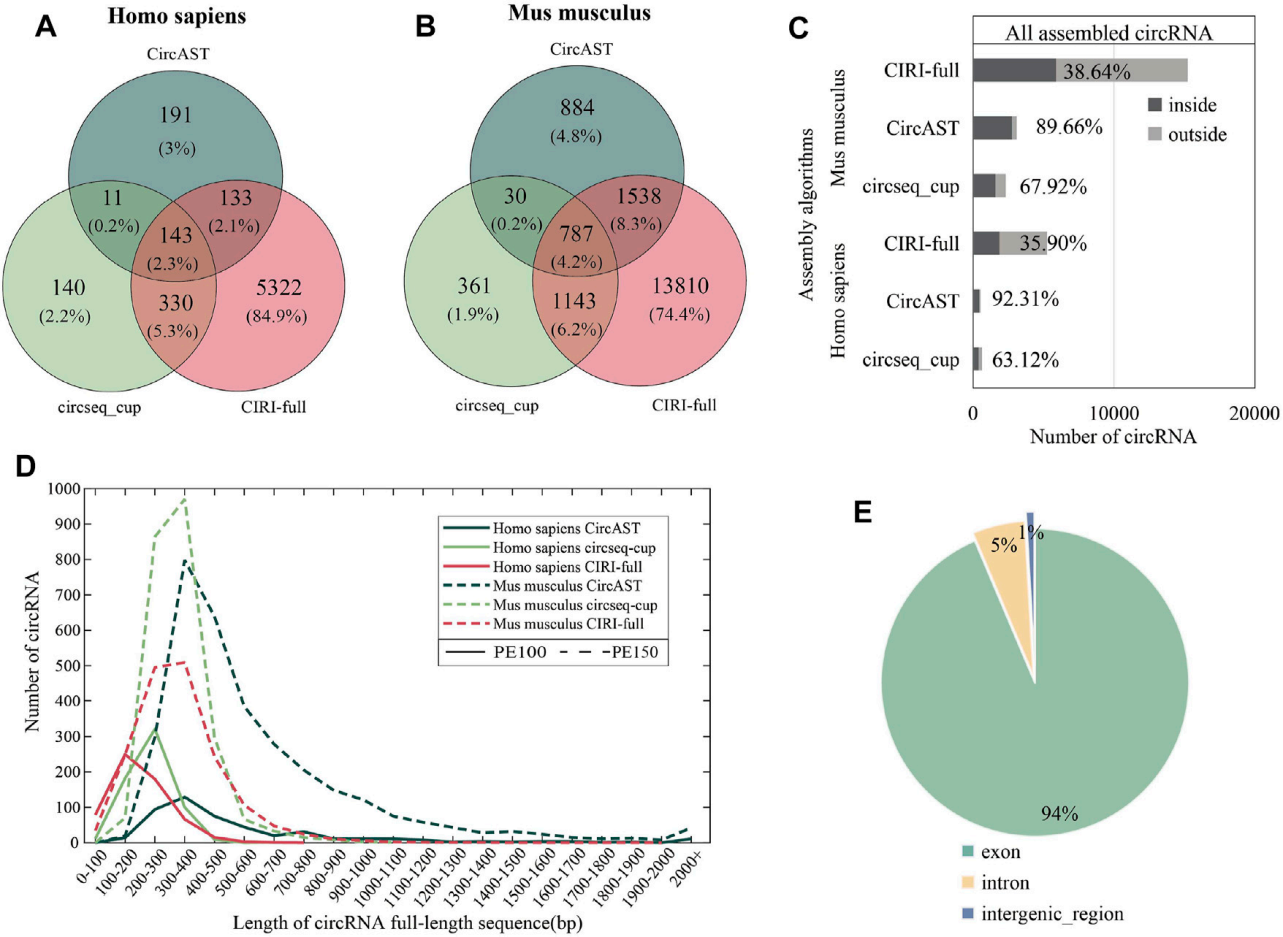
Assembly results of three assembly tools. **(A,B)** Venn diagram depicting the overlap between different assembly algorithms in human and mouse datasets. **(C)** The proportion of full-length circRNAs constructed from the common circRNA candidates. ‟inside” (dark gray) represents assembled circRNAs belonging to common circRNAs among four identification tools, and ‘outside’ (light gray) represents assembled circRNAs not belonging to common circRNA among four identification tools. **(D)** Length distribution of circRNA full-length sequences (the result of CIRI-full is scaled by 1/10). **(E)** The percentage of circRNA categories in all assembled circRNA results.

Among three assembly tools, full-length circRNAs assembled using CIRI-full were more than those assembled using CircAST and circseq_cup. For example, for the circRNA identification result of CIRI on sample SRR10612068, 300 (6.21%) and 1868 (38.69%) full-length circRNAs were assembled using CircAST and CIRI-full, whereas circsesq_cup identified 323 full-length circRNAs for sample SRR10612068 (Table 1 and Supplementary Table S2). In addition, some unique circRNAs that were only identified using a single circRNA identification algorithm were reconstructed successfully (Supplementary Figure S1), indicating that the selection of circRNA identification software had impact on CircAST and CIRI-full. Using CIRI as a circRNA identification tool, CircAST and CIRI-full generated more circRNA full-length sequences than other identification tools (CIRCexplorer, circRNA_finder, and find_circ). For common circRNA candidates in four circRNA identification algorithms, most full-length circRNAs (60%–90%) produced by CircAST and circseq_cup were constructed from the common candidates, while less than half of full-length circRNAs by CIRI-full were involved in common candidates (Figure 3C). It was found that the lengths of most full-length circRNAs were shorter than 1,000 bp (Figure 3D). CircAST can assemble longer sequences for human and mouse, which is consistent with the advantage of CircAST that it can assemble long circRNAs without using long sequencing reads. However, the performance of CIRI-full was not consistent in PE100 and PE150 (Figure 3D). Origin also is an important factor in reconstructing full-length sequences; most full-length circRNAs (94%) were derived from the exon region on the genome in our results (Figure 3E), which can be explained by the following: first, more than 90% circRNA candidates belong to exonic circRNAs and second, exonic circRNAs were usually supported by more back splice reads.

**TABLE 1:** Assembly rate and assembly number of circRNA using different assembly tools.

	CircAST^a^				CIRI-full^a^				circseq_cup^a^
	CIRI^b^	CIRCexplorer^b^	circRNA_finder^b^	find_circ^b^	CIRI^b^	CIRCexplorer^b^	circRNA_finder^b^	find_circ^b^	
SRR10612068	300 (6.21%)	129 (3.98%)	128 (3.80%)	248 (4.86%)	1868 (38.69%)	1,121 (34.61%)	1,131 (33.56%)	1,661 (32.55%)	323
SRR10612069	256 (5.95%)	96 (3.71%)	95 (3.55%)	201 (4.51%)	1723 (40.03%)	948 (36.66%)	967 (36.11%)	1,452 (32.56%)	286
SRR10612070	259 (5.99%	111 (3.98%)	96 (3.37%)	204 (4.37%)	1723 (39.85%	950 (34.10%)	940 (33.01%)	1,508 (32.31%)	285
CRR194214	1958 (16.15%)	1,254 (11.64%)	1,155 (9.92%)	1,292 (10.81%)	7,353 (60.64%)	5,410 (50.23%)	5,658 (48.61%)	5,919 (49.54%)	1,509
CRR194215	2,724 (19.87%)	1852 (13.91%)	1706 (11.55%)	1769 (11.99%)	8,480 (61.86%)	6,526 (49.02%)	6,923 (46.89%	7,095 (48.10%)	1847

The table displays the number of full-length circRNA, and the assembly rate for CircAST, and CIRI-full (The numbers in parenthesis is the assembly rate); and the last column displays the number of full-length circRNA, for circseq_cup. The superscript ‘a’ indicates that the term is an assembly tool, and superscript ‘b’ indicates that the term is a identification algorithm. Assembly rate = A/I, where A is number of assembled circRNA, I is number of all identified circRNA.

### Evaluation of Different Sequence Assembly Strategies From Short Reads

There are three assembly tools for assembly of circRNA full-length sequences from short reads, but it is unknown which one has the best performance. Here, we used three evaluation strategies (read alignment, CIRI-long, and isoCirc) to evaluate the performance of nine assembly strategies due to different combinations of circRNA identification software (CIRI, CIRIexplorer, circRNA_finder, and find_circ) and assembly tools (circseq_cup, CIRI_full, and CircAST).

**FIGURE 4:**
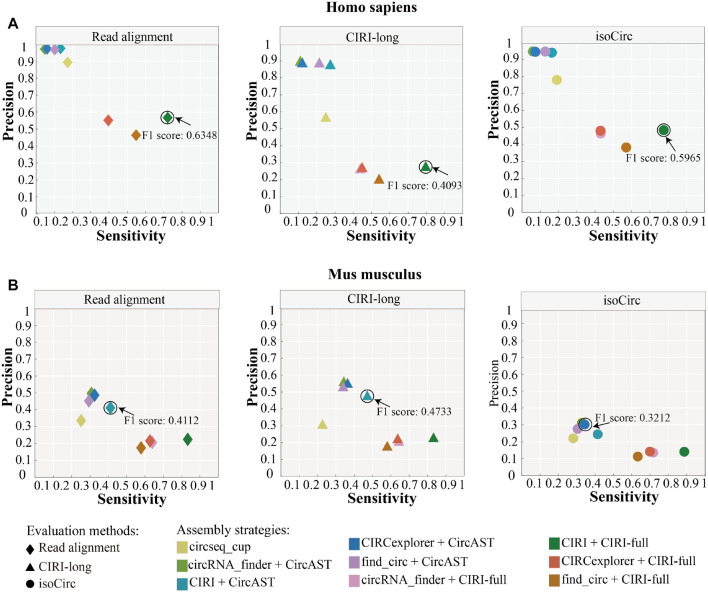
Performance of different assembly strategies in terms of sensitivity and precision. Marked points are the best assembly strategy under different evaluation methods. **(A)** *Homo sapiens.* **(B)** *Mus musculus.*

As shown in Figure 4, circseq_cup showed different precision (56.57–89.26%) when evaluated using different evaluation strategies in human datasets and lower than 30% sensitivity. In mouse datasets, circseq_cup showed lower precision and sensitivity. For human datasets, CircAST achieved precision higher than 85% and sensitivity lower than 30%, and CIRI-full gained precision lower than 60% and sensitivity higher than 39%. CircAST and CIRI-full showed the same trend in mouse datasets. circseq_cup and CircAST showed high precision and low sensitivity whereas CIRI-full displayed low precision and high sensitivity. It is feasible to improve the precision at the cost of sensitivity for CIRI-full.

In addition, the assembly strategy of CIRI plus CIRI-full showed the highest F1 score (read alignment: 0.6348, CIRI-long: 0.4093, and isoCirc: 0.5965) using all three evaluation strategies in human datasets (Figure 4, Supplementary Table S3). However, CircAST performed better than CIRI-full in mouse datasets. For mouse datasets, using read alignment and CIRI-long as evaluation strategies, the combination of CIRI and CircAST showed the highest F1 score (read alignment: 0.4112, CIRI-long: 0.4733), and the combination of CIRCexplorer and CircAST produced the highest F1 score (0.3212) when using isoCirc as the evaluation strategy. Overall, CIRI-full showed better performance for human datasets, and CircAST showed better performance for mouse datasets.

### Comparison of Evaluation Strategies

As shown in Figure 1, three evaluation strategies (see the Method section) were used to evaluate circRNA full-length sequence assembly using long reads.

In Supplementary Figure S2, for each evaluation strategy, we combined all positive datasets (full-length circRNAs that were verified correctly) of nine assembly strategies to compare the evaluation strategies. Of all correct full-length circRNAs in human datasets, 1,337 full-length circRNAs (39.1%) were observed between all evaluation strategies, and read alignment confirmed 3,217 full-length circRNA that accounted for about 94% of all verified results (Supplementary Figure S2A). Similarly, there were 1,391 (34.9%) verified full-length circRNAs found in the results of all three evaluation strategies in mouse datasets. For mouse datasets, instead of read alignment, CIRI-long generated the largest number of verified circRNA sequences (3,128, 78.5%) (Supplementary Figure S2B).

Then, we compared precision of nine assembly strategies under three evaluation methods. In human datasets, read alignment showed the highest precision for all nine assembly strategies, while for mouse datasets, CIRI-long showed the highest precision for eight assembly strategies (Supplementary Figure S2C,D). Evaluation strategies showed various performances in human and mouse datasets. The precision of CIRI-long was higher than that of isoCirc for human datasets, while for mouse datasets, the opposite trend was observed.

To analyze the reason for the opposite trend observed between CIRI-long and isoCirc, we generated five subset samples from SRR10612050 according to read length (<1,000 bp, 2000–2,300 bp, 3,500–3,530 bp, 5,000–5,050 bp, and 6,900–7,000 bp) (Table S4). The majority of circRNAs were identified by CIRI-long for read lengths less than 1,000 bp, and isoCirc identified more circRNAs when read length was longer than 1,000 bp. The results showed that CIRI-long and isoCirc tend to behave differently for different read lengths.

From the above analysis, it was found that using circRNA sequences that are verified by all three evaluation methods are more reliable; however, in order to generate enough number of circRNA sequences, we chose to use the circRNA sequences verified by at least two of the three evaluation strategies. In the flowing analysis, we combined all the correct full-length circRNAs verified by at least two evaluation strategies.

### Number of Back Splice Reads Affects the Quality of Reconstructed circRNA Sequences

It is found that the circRNA assembly results of circseq_cup and CircAST displayed higher precision than CIRI-full, whereas CIRI-full displayed the highest sensitivity. In this part, we analyzed the impact of the back splice reads on the precision of creditable full-length circRNAs which were verified by at least two evaluation methods.

Supplementary Figure S3 illustrates the change of precision of assembly tools with the increasing number of back splice reads given in human datasets. With the increasing number of back splice reads, the precision of circseq_cup and CIRI-full were also increased. However, the precision of CircAST did not show a similar trend (Supplementary Figure S3). The curves of CircAST showed larger fluctuations due to its low sensitivity, and a lower number of wrong circRNAs causes a sharp decrease in precision. In mouse datasets, the precision of all assembly strategies increased with the increasing number of back splice reads (Supplementary Figure S4). We can assemble more reliable full-length sequences when circRNAs were supported by many back splice reads.

### Improving circRNA Sequence Assembly for CIRI-Full

Previous results showed that for human datasets, circseq_cup and CircAST assembled a lower number of circRNA sequences with high precision and low sensitivities, and most of them (∼80%) were verified as correct. Meanwhile, CIRI-full generated more full-length sequences of circRNAs, and only less than 57% of circRNA sequences were evaluated as correct. Therefore, one can improve the precision by screening more credible sequences at the cost of sensitivity.

**FIGURE 5:**
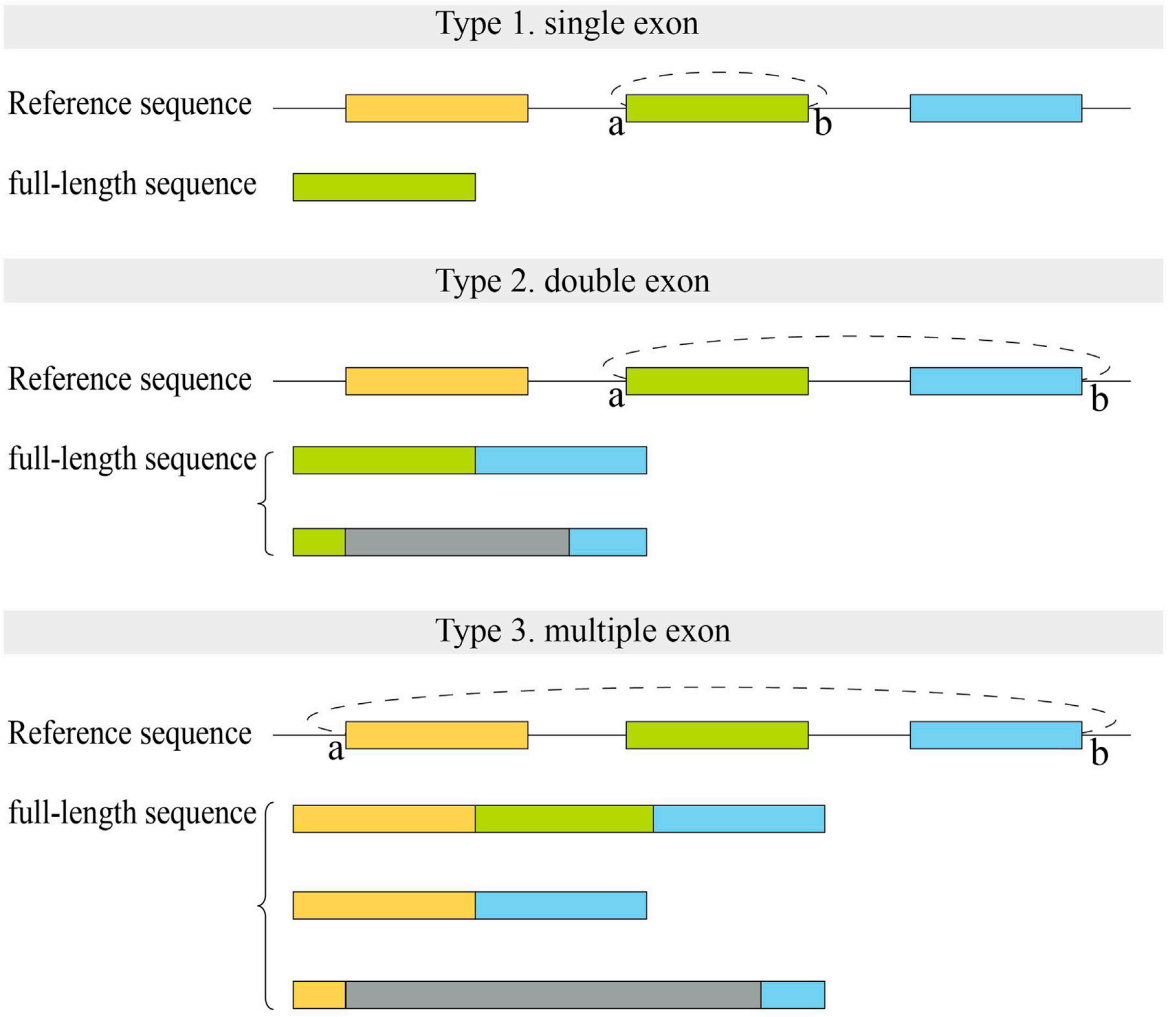
Structure of full-length sequences reconstructed by CIRI-full in human datasets. Small letters **(A,B)** represent two back splice sites. Color rectangles represent exons, and gray rectangles represent the uncertain region which may include exons or introns.

We first analyzed the sequences of exonic full-length circRNAs in CIRI-full for human datasets (Figure 5). For full-length circRNAs that were derived from a single exon, more than 90% of circRNA full-length sequences were full exon sequences in assembly results (Type 1). In the reconstructed results of circRNAs derived from two adjacent exons, about 40–50% of sequences contained two complete exons with no intron sequences (Type 2). Fewer (∼16%) full-length circRNAs derived from multiple exons consisted of all exon sequences between back splice sites (Type 3).

**FIGURE 6:**
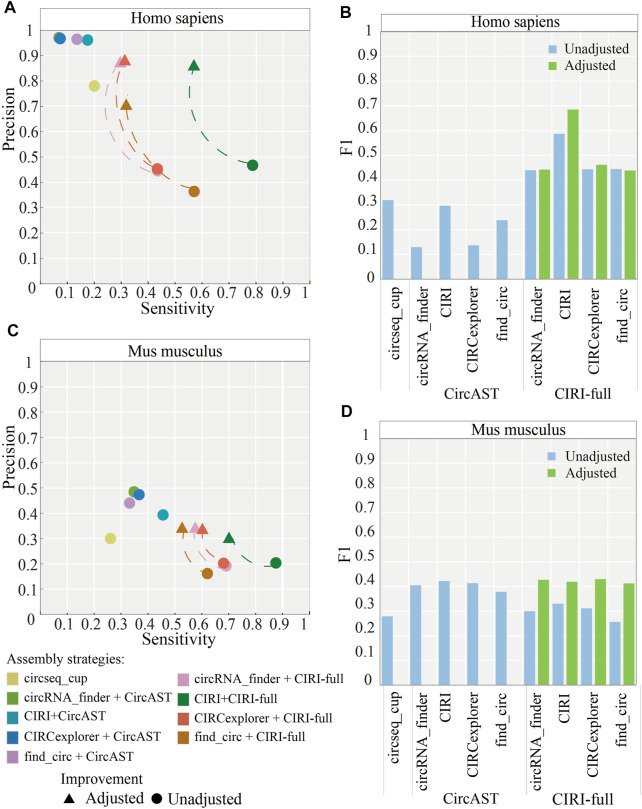
Performance of assembly strategies related to CIRI-full after adjustment (screening). **(A,B)** Performance of assembly strategies in human and mouse datasets. **(C,D)** F1 score of assembly strategies in human and mouse datasets. “Adjusted” represents performance of CIRI-full after screening and “Unadjusted” represents performance of CIRI-full before screening.

In addition, we calculated the ratio between full-length circRNAs that consisted of all exon back splice sites from CIRI-full and the correct ones. It was found that more than 80% of full-length sequences consisting of all exons between back splice sites were verified correctly. Thus, to improve the precision of CIRI-full, we screened exonic circRNA that full-length sequences consisted of all exon sequences between back splice sites; these sequences were considered more reliable and were selected as correct sequences. After applying the screening protocol, the average precision of CIRI-full over four circRNA identification algorithms increased from 43.26 to 82.77% in human datasets (Figure 6A), and the average F1 score increased from 0.4788 to 0.5069 (Figure 6C).

The same screening rule was also applied in the mouse datasets; the average precision of CIRI-full over four circRNA identification algorithms increased from 18.96 to 32.82% (Figure 6B), and the average F1 score increased from 0.2995 to 0.4223 (Figure 6D). CIRI-full showed higher F1 score than CircAST in mouse datasets after screening.

## Discussion

Reconstruction of circRNA full-length sequences is vital for its function identification. Three assembly tools were developed to assemble full-length sequences using short reads, and two of them, CircAST and CIRI-full, require identification information of circRNA to complete assembly.

Here, we calculated the assembly rate of CircAST and CIRI-full in all datasets and the number of full-length circRNAs on circseq_cup (Table 1). For the same sample, CIRI-full produced more circRNAs full-length sequences than CircAST and circseq_cup.

As we know, in addition to BSJ, CIRI-full also proposed a new feature, named RO (Y. Zheng et al., 2019). The combination of BSJ and RO could assemble full-length sequences of some circRNAs, these circRNAs lacking support reads on internal sequences when they were assembled only using BSJ. Besides, incomplete full-length sequences were also included in the results. Thus, CIRI-full had the highest sensitivity and lowest precision among the three assembly tools (Figure 4). CircAST and circseq_cup chose another way and provided full-length sequences with high precision (Wu et al., 2019). CircAST had a low assembly rate due to filtered out circRNAs that were supported by less than 12 back splice reads. circseq_cup screened reliable back splice reads by several criteria to ensure the correctness of full-length sequences. High precision and sensitivity are our ultimate goal. In this study, we screened some circRNA full-length sequences that consisted of all exons between back splice sites in CIRI-full as final results. This procedure increased the precision and F1 score of CIRI-full (Figure 6).

In addition, as shown in Table 1, assembly tools displayed higher assembly rate in mouse than human, whereas assembly tools displayed poor performance in mouse datasets when we evaluated the performance using three evaluation strategies based on long reads (Supplementary Table S3). High assembly rate in mouse datasets is due to the feature of short reads. Short reads of mouse had bigger sequencing depth and longer sequence reads than human datasets (Supplementary Table S1) (X. Li et al., 2020). The number and length of back splice reads affect the assembly rate of assembly tools. Mouse datasets find it easier to assemble more circRNA full-length sequences than human datasets. Evaluation of performance was based on corresponding long reads in this study. For short reads of mouse, long reads datasets and short reads are not matched perfectly. The small long reads datasets lead to only part of full-length sequences that could be verified. Big short reads datasets and small long reads datasets make assembly tools show poor performance and low precision and sensitivity.

As shown in Figure 6A and Figure 6C, the precision of CIRI-full is improved by about 40% in human datasets and about 10% in mouse datasets. The difference was caused by sequencing datasets. The size of short reads and long reads are similar in human datasets; long reads could be used to verify most candidate circRNAs. By removing part of low-confidence full length circRNAs, the precision of CIRI-full was greatly improved. The short reads data are much bigger than long reads in mouse datasets; thus, only a small part of candidate circRNAs was verified by the long reads, and the precision of CIRI-full for mouse datasets was not improved as much as for human datasets.

This work indicated that the combination of CIRI and CIRI-full is a better assembly strategy for the single assembly algorithm, and several reported assembly tools should be used simultaneously to obtain comprehensive and reliable results. However, we only used two datasets (in human and mouse) to evaluate the performance of assembly tools, and human and mouse are both mammals. Thus, our conclusion is more applicable to mammals, and whether it is applicable to other animals or plants still needs further verification. In addition, developing a new assembly algorithm that has the advantages of lower data requirements and more reliable assembly results is more significant.

## Data Availability

Publicly available datasets were analyzed in this study. This data can be found here: https://bigd.big.ac.cn/gsa https://www.ncbi.nlm.nih.gov/sra.
