# Corrigendum: Evaluation of CircRNA sequence assembly methods using long reads

**DOI:** 10.3389/fgene.2023.1248519

**Published:** 2023-07-06

**Authors:** Jingjing Zhang, Md. Tofazzal Hossain, Weiguo Liu, Yin Peng, Yi Pan, Yanjie Wei

**Affiliations:** ^1^ University of Chinese Academy of Sciences, Beijing, China; ^2^ Centre for High Performance Computing, Shenzhen Institutes of Advanced Technology, Chinese Academy of Sciences, Shenzhen, China; ^3^ School of Software, Shandong University, Jinan, China; ^4^ Guangdong Key Laboratory for Genome Stability and Disease Prevention and Regional Immunity and Diseases, Department of Pathology, School of Medicine, Shenzhen University, Shenzhen, China; ^5^ CAS Key Laboratory of Health Informatics, Shenzhen Institutes of Advanced Technology, Chinese Academy of Sciences, Shenzhen, China

**Keywords:** circRNA, full-length sequences, short reads, long reads, assembly

In the published article, there was an error in the **Funding** statement. The grant number statement for the Shenzhen Basic Research Fund was displayed as “RCYX2020071411473419”. The correct grant number is “RCYX20200714114734194”. The correct Funding statement appears below.

“This work was partly supported by the National Key Research and Development Program of China under grant No. 2018YFB0204403, the Strategic Priority CAS Project XDB38050100, the National Science Foundation of China under grant no. U1813203, the National Natural Youth Science Foundation of China under grant no. 31601028, the Shenzhen Basic Research Fund under grant nos. JCYJ20190808163801777, KQTD20200820113106007, and RCYX20200714114734194, and the CAS Key Lab under grant no. 2011DP173015.”

The authors apologize for this error and state that this does not change the scientific conclusions of the article in any way. The original article has been updated.

