# Supplementary material for: Evaluation of CircRNA Sequence Assembly Methods Using Long Reads
Source: Front Genet. 2022 Feb 14;13:816825. doi: 10.3389/fgene.2022.816825 (PMC8882733; doi:10.3389/fgene.2022.816825)
Supplement: Supplementary file 1 [file DataSheet1.docx]

Supplementary Material

**Supplementary Table 1.** Summary of the datasets used in this study.

|  | [Platform](https://www.ncbi.nlm.nih.gov/Traces/study/?acc=SRP235284&o=acc_s:a) | [LibraryLayout](https://www.ncbi.nlm.nih.gov/Traces/study/?acc=SRP235284&o=acc_s:a) | Reads length(bp) | Reads count | Organism |
| --- | --- | --- | --- | --- | --- |
| SRR10612068 | Illumina HiSeq 2500 | PAIRED | 101 | 70284760*2 | *Homo sapiens* |
| SRR10612069 | Illumina HiSeq 2500 | PAIRED | 101 | 56542019*2 | *Homo sapiens* |
| SRR10612070 | Illumina HiSeq 2500 | PAIRED | 101 | 62545682*2 | *Homo sapiens* |
| SRR10612050 | OXFORD_NANOPORE (MinION) | SINGLE | 4395 | 2826759 | *Homo sapiens* |
| SRR10612051 | OXFORD_NANOPORE (MinION) | SINGLE | 4644 | 3023085 | *Homo sapiens* |
| SRR10612052 | OXFORD_NANOPORE (MinION) | SINGLE | 4364 | 4236848 | *Homo sapiens* |
| SRR10612053 | OXFORD_NANOPORE (MinION) | SINGLE | 4737 | 3767164 | *Homo sapiens* |
| SRR10612054 | OXFORD_NANOPORE (MinION) | SINGLE | 4672 | 3816000 | *Homo sapiens* |
| SRR10612055 | OXFORD_NANOPORE (MinION) | SINGLE | 4769 | 2649639 | *Homo sapiens* |
| CRR194214 | Illumina HiSeq X Ten | PAIRED | 151 | 72803363*2 | *Mus musculus* |
| CRR194215 | Illumina HiSeq X Ten | PAIRED | 151 | 92170218*2 | *Mus musculus* |
| CRR194190 | OXFORD_NANOPORE MinION | SINGLE | 400,600,1000 | 3429608 | *Mus musculus* |
| CRR194191 | OXFORD_NANOPORE MinION | SINGLE | 400,600,1000 | 2399427 | *Mus musculus* |
| CRR194194 | OXFORD_NANOPORE MinION | SINGLE | 400,600,1000 | 4631205 | *Mus musculus* |
| CRR194195 | OXFORD_NANOPORE MinION | SINGLE | 400,600,1000 | 2259493 | *Mus musculus* |

**Supplementary Table 2.** Number of circRNA identified by different assembly tools based on short reads.

|  | CIRI2 | CIRCexplorer2 | circRNA_finder | find_circ |
| --- | --- | --- | --- | --- |
| SRR10612068 | 4828 | 3239 | 3370 | 5103 |
| SRR10612069 | 4304 | 2586 | 2678 | 4460 |
| SRR10612070 | 4324 | 2786 | 2848 | 4667 |
| CRR194214 | 12126 | 10770 | 11639 | 11947 |
| CRR194215 | 13708 | 13313 | 14765 | 14752 |


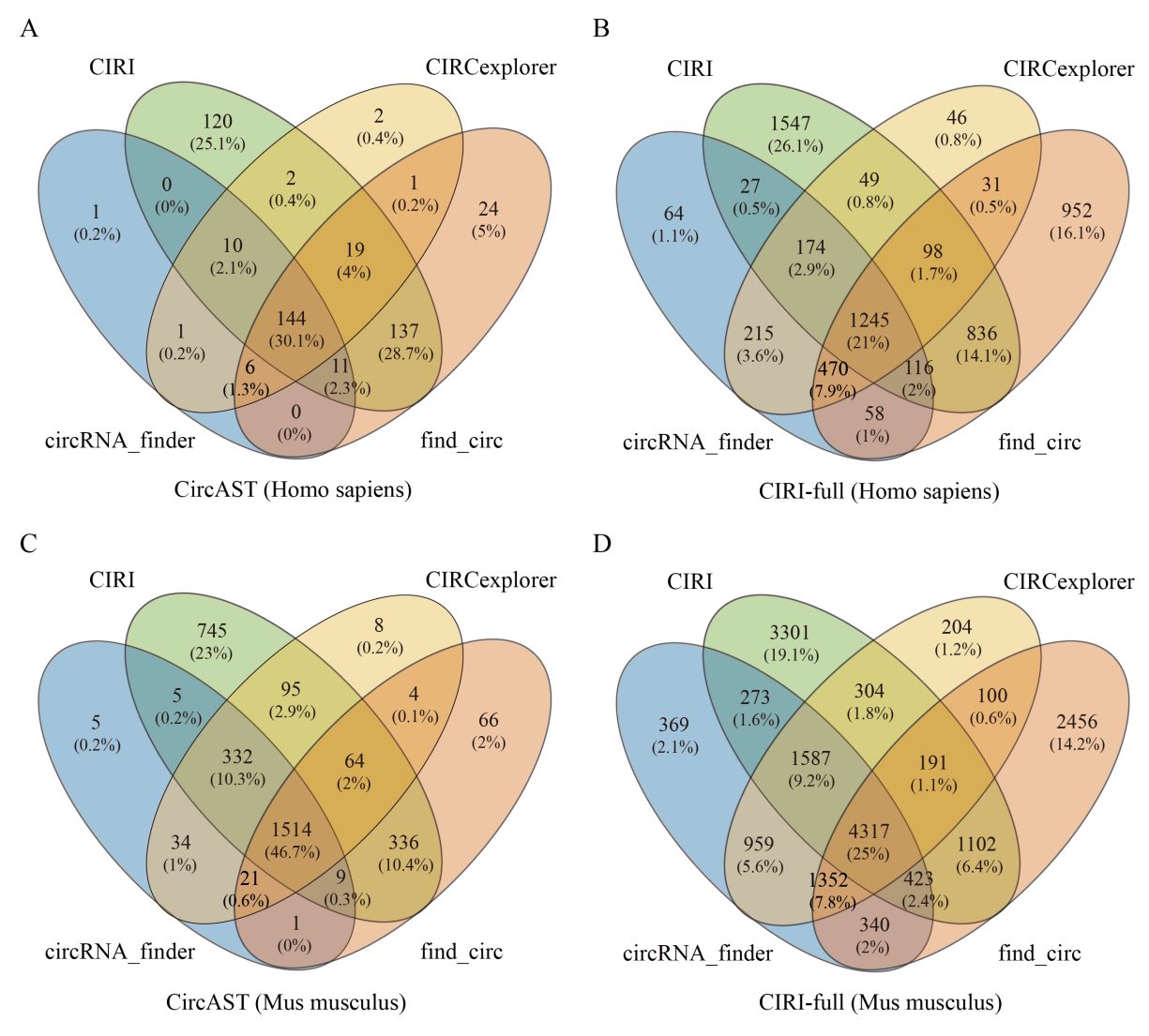


**Supplementary Figure 1.** Venn diagram depicting the overlap between the assembly results of different identification algorithms based on the short reads of the human and mouse datasets.

**Supplementary Table 3.** Summary of accuracy measures in the human and mouse datasets.

| **Read alignment** | Homo sapiens | | | | | Mus musculus | | | | |
| --- | --- | --- | --- | --- | --- | --- | --- | --- | --- | --- |
| Methods | #Assembled | TP | P% | S% | F1 | #Assembled | TP | P% | S% | F1 |
| Circseq_cup | 624 | 557 | 89.26% | 17.31% | 0.2900 | 2325 | 780 | 33.55% | 25.19% | 0.2878 |
| CircRNA_finder=>CircAST | 173 | 168 | 97.11% | 5.22% | 0.0991 | 1921 | 954 | 49.66% | 30.81% | 0.3803 |
| CIRI=>CircAST | 443 | 432 | 97.52% | 13.43% | 0.2361 | 3100 | 1274 | 41.10% | 41.14% | **0.4112** |
| CIRCexplorer=>CircAST | 185 | 180 | 97.30% | 5.60% | 0.1058 | 2072 | 1007 | 48.60% | 32.52% | 0.3897 |
| Find_circ=>CircAST | 342 | 331 | 96.78% | 10.29% | 0.1860 | 2015 | 911 | 45.21% | 29.42% | 0.3565 |
| CircRNA_finder=>CIRI-full | 2369 | 1277 | 53.90% | 39.70% | 0.4572 | 9622 | 1982 | 20.60% | 64.02% | 0.3117 |
| CIRI=>CIRI-full | 4092 | 2320 | 56.70% | 72.12% | **0.6348** | 11500 | 2580 | 22.43% | 83.33% | 0.3535 |
| CIRCexplorer=>CIRI-full | 2328 | 1273 | 54.68% | 39.57% | 0.4592 | 9015 | 1942 | 21.54% | 62.73 | 0.3207 |
| Find_circ=>CIRI-full | 3806 | 1764 | 46.35% | 54.83% | 0.5023 | 10282 | 1792 | 17.43% | 57.88 | 0.2679 |
| **CIRI-long** | Homo sapiens | | | | | Mus musculus | | | | |
| Methods | #Assembled | TP | P% | S% | F1 | #Assembled | TP | P% | S% | F1 |
| Circseq_cup | 624 | 353 | 56.57% | 24.95% | 0.3462 | 2325 | 711 | 30.58% | 22.73% | 0.2608 |
| CircRNA_finder=>CircAST | 173 | 155 | 89.60% | 10.95% | 0.1952 | 1921 | 1074 | 55.91% | 34.34% | 0.42548 |
| CIRI=>CircAST | 443 | 388 | 87.58% | 27.42% | 0.4176 | 3100 | 1474 | 47.55% | 47.12% | **0.4733** |
| CIRCexplorer=>CircAST | 185 | 164 | 88.65% | 11.59% | 0.205 | 2072 | 1138 | 54.92% | 36.38% | 0.4377 |
| Find_circ=>CircAST | 342 | 303 | 88.60% | 21.41% | 0.3449 | 2015 | 1063 | 52.75% | 33.98% | 0.4134 |
| CircRNA_finder=>CIRI-full | 2369 | 620 | 26.17% | 43.82% | 0.3277 | 9622 | 2012 | 20.91% | 64.32% | 0.3156 |
| CIRI=>CIRI-full | 4092 | 1127 | 27.54% | 79.65% | **0.4093** | 11500 | 2605 | 22.65% | 83.28% | 0.3562 |
| CIRCexplorer=>CIRI-full | 2328 | 628 | 26.98% | 44.38% | 0.3356 | 9015 | 1994 | 22.12% | 63.75% | 0.3284 |
| Find_circ=>CIRI-full | 3806 | 766 | 20.13% | 54.13% | 0.2934 | 10282 | 1815 | 17.65% | 58.02% | 0.2707 |
| **isoCirc** | Homo sapiens | | | | | Mus musculus | | | | |
| Methods | #Assembled | TP | P% | S% | F1 | #Assembled | TP | P% | S% | F1 |
| Circseq_cup | 624 | 487 | 78.04% | 19.08% | 0.3066 | 2325 | 513 | 22.06% | 28.09% | 0.2472 |
| CircRNA_finder=>CircAST | 173 | 164 | 94.80% | 6.42% | 0.1203 | 1921 | 599 | 31.18% | 32.80% | 0.3197 |
| CIRI=>CircAST | 443 | 417 | 94.13% | 16.33% | 0.2784 | 3100 | 757 | 24.42% | 41.46% | 0.3073 |
| CIRCexplorer=>CircAST | 185 | 175 | 94.59% | 6.85% | 0.1278 | 2072 | 626 | 30.21% | 34.28% | **0.3212** |
| Find_circ=>CircAST | 342 | 324 | 94.74% | 12.69% | 0.2238 | 2015 | 556 | 27.59% | 30.45% | 0.2895 |
| CircRNA_finder=>CIRI-full | 2369 | 1100 | 46.43% | 43.09% | 0.4470 | 9622 | 1302 | 13.53% | 71.30% | 0.2275 |
| CIRI=>CIRI-full | 4092 | 1982 | 48.44% | 77.63% | **0.5965** | 11500 | 1619 | 14.08% | 88.66% | 0.2430 |
| CIRCexplorer=>CIRI-full | 2328 | 1098 | 47.16% | 43.01% | 0.4499 | 9015 | 1277 | 14.17% | 69.93% | 0.2356 |
| Find_circ=>CIRI-full | 3806 | 1457 | 38.28% | 57.07% | 0.4582 | 10282 | 1156 | 11.24% | 63.31% | 0.1909 |

Note: The total number of full-length circRNAs and true positives predicted by each assembly strategies were calculated. #Assembled, the number of assembled circRNA full-length sequences; TP, true positives; P, precision; S, sensitivity; F1, F1 score.


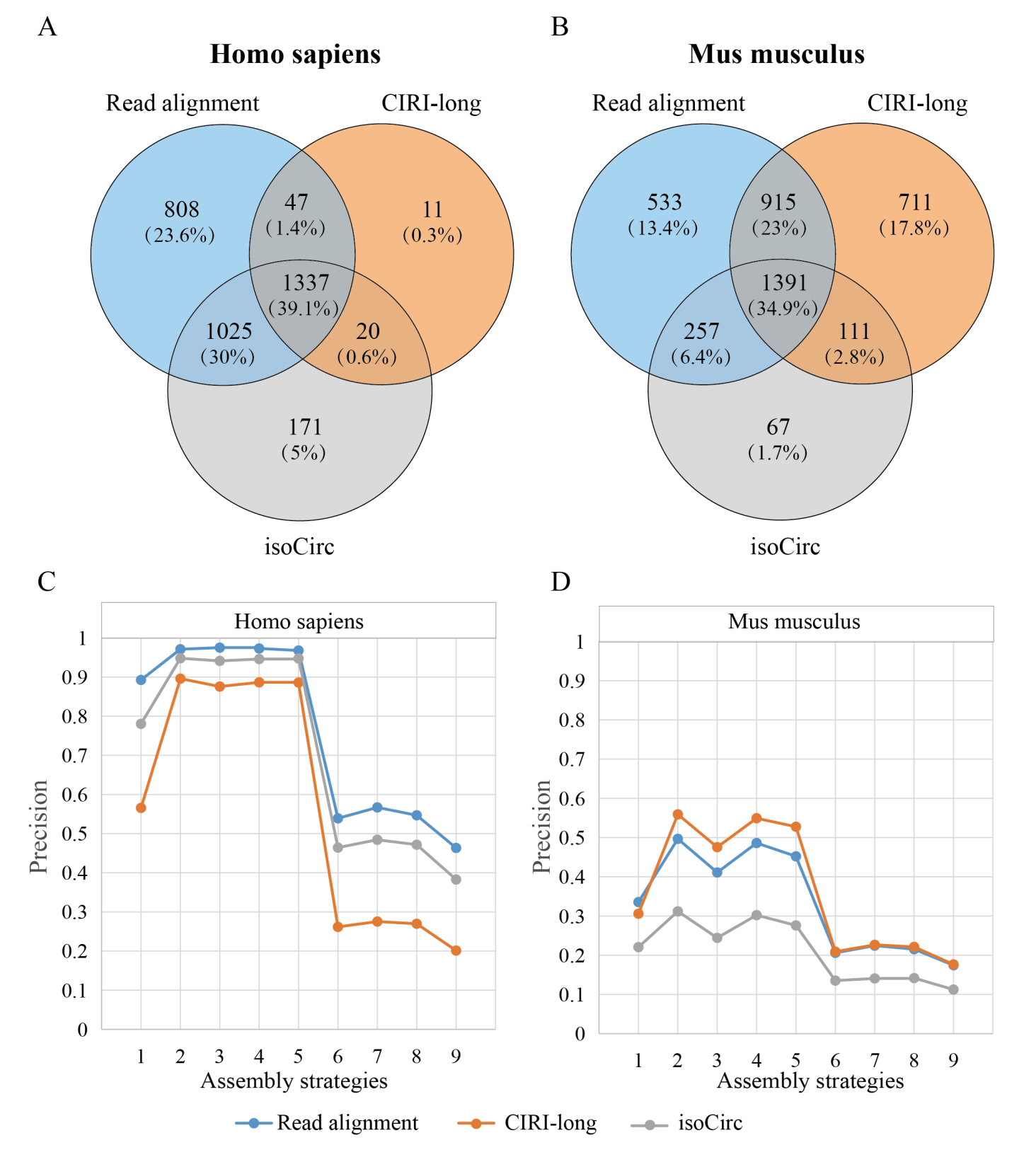


**Supplementary Figure 2.** Comparison of evaluation strategies. (A and B) Venn diagram depicting the overlap between different evaluation strategies in human and mouse datasets. (C and D) Precision of assembly strategies when using three evaluation strategies. Digital representation assembly strategies of horizontal axis. 1, circseq_cup; 2, circRNA_finder + CircAST; 3, CIRI + CircAST; 4, CIRCexplorer + CircAST; 5, find_circ + CircAST; 6, circRNA_finder + CIRI-full; 7, CIRI + CIRI-full; 8, CIRCexplorer + CIRI-full; 9, find_circ +CIRI-full.

**Supplementary Table 3.** Results of CIRI-long and isoCirc on the subsets of SRR10612050 datasets with different read lengths.

|  | Length of reads | Size of data (Mb) | #circRNA (CIRI-long) | #circRNA (isoCirc) |
| --- | --- | --- | --- | --- |
| Data A | < 1000 bp | 178 | 792 | 76 |
| Data B | 2000-2300 bp | 186 | 180 | 815 |
| Data C | 3500-3530 bp | 206.2 | 142 | 942 |
| Data D | 5000-5050 bp | 195 | 47 | 731 |
| Data E | 6900-7000 bp | 209 | 14 | 675 |

Note: Column 4 and 5 represent the number of identified circRNAs using CIRI-long and isoCirc with differnt read


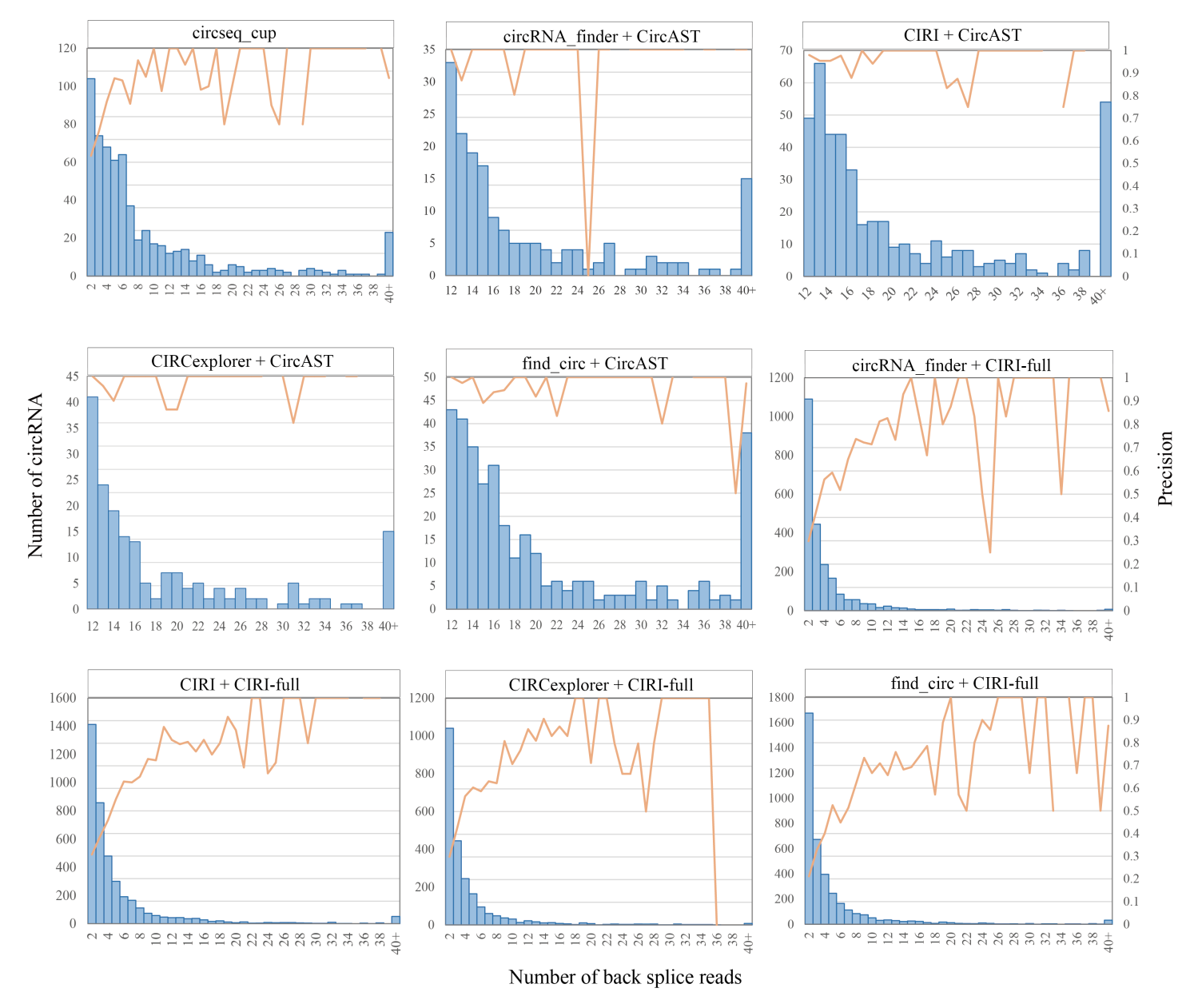


**Supplementary Figure 3.** Impact of back splice reads on the assembly precision in human datasets. Histogram represents is the distribution of full-length circRNAs as back splice reads increase, solid orange lines represent the precision of assembly strategies at different number of back splice reads.


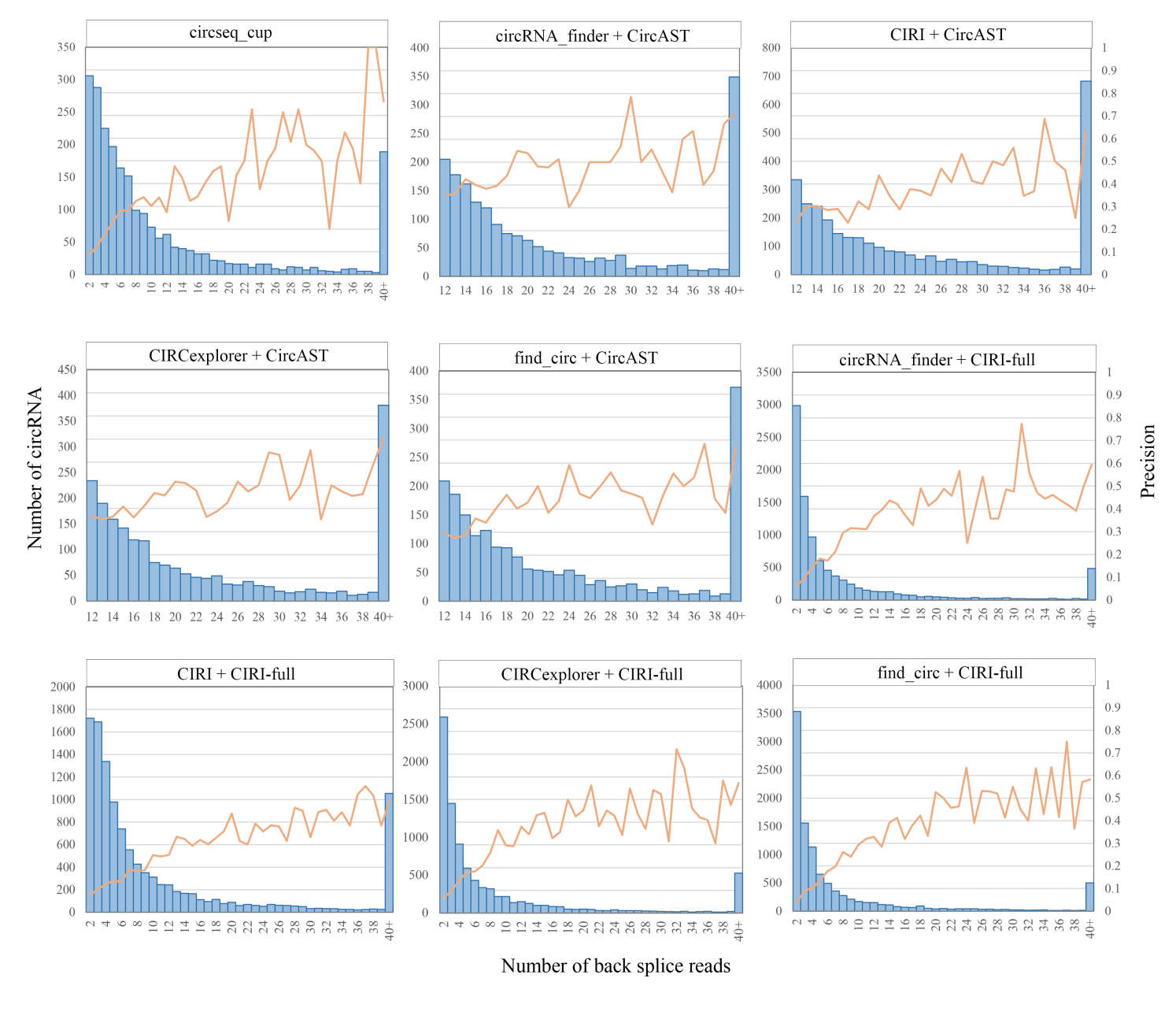


**Supplementary Figure 3.** Impact of back splice reads on the assembly precision in mouse datasets. Histogram represents is the distribution of full-length circRNAs as back splice reads increase, solid orange lines represent the precision of assembly strategies at different number of back splice reads.
